# Characterization of an Isostructural MOF Series of Imidazolate Frameworks Potsdam by Means of Sorption Experiments with Water Vapor

**DOI:** 10.3390/nano11061400

**Published:** 2021-05-25

**Authors:** Dirk Otter, Suvendu Sekhar Mondal, Anas Alrefai, Lorenz Krätz, Hans-Jürgen Holdt, Hans-Jörg Bart

**Affiliations:** 1 Lehrstuhl für Thermische Verfahrenstechnik, Technische Universität Kaiserslautern, Gottlieb-Daimler-Straße 44, 67663 Kaiserslautern, Germany; dirk.otter@mv.uni-kl.de (D.O.); kraetz@mv.uni-kl.de (L.K.); 2 Institut für Chemie, Anorganische Chemie, Universität Potsdam, Karl-Liebknecht-Straße 24-25, 14476 Potsdam, Germany; suvendu.iitb@gmail.com (S.S.M.); alrefai@uni-potsdam.de (A.A.); holdt@uni-potsdam.de (H.-J.H.)

**Keywords:** material characterization, water vapor, adsorption, hysteresis, Imidazolate frameworks Potsdam

## Abstract

Sorption measurements of water vapor on an isoreticular series of Imidazolate Frameworks Potsdam (IFP), based on penta-coordinated metal centers with secondary building units (SBUs) connected by multidentate amido-imidate-imidazolate linkers, have been carried out at 303.15 K. The isotherm shapes were analyzed in order to gain insight into material properties and compared to sorption experiments with nitrogen at 77.4 K and carbon dioxide at 273.15 K. Results show that water vapor sorption measurements are strongly influenced by the pore size distribution while having a distinct hysteresis loop between the adsorption and desorption branch in common. Thus, IFP-4 and -8, which solely contain micropores, exhibit H4 (type I) isotherm shapes, while those of IFP-1, -2 and -5, which also contain mesopores, are of H3 (type IV) shape with three inflection points. The choice of the used linker substituents and transition metals employed in the framework has a tremendous effect on the material properties and functionality. The water uptake capacities of the examined IFPs are ranging 0.48 mmol g^−1^ (IFP-4) to 6.99 mmol g^−1^ (IFP-5) and comparable to those documented for ZIFs. The water vapor stability of IFPs is high, with the exception of IFP-8.

## 1. Introduction

Due to their modular nature, metal organic frameworks (MOFs) offer a wide variety of properties such as pore geometry and functionality, which make them particularly attractive for the use in adsorption-based separation processes [1,2,3] and heterogeneous catalysis [4,5]. A major drawback of these species, however, is their often documented susceptibility towards water [6,7,8,9]. Therefore, there is a great interest in building a deeper understanding of the interaction of MOF-species with water. Valuable reviews dedicated to this topic were recently provided by the research groups of Walton [10] and Farrusseng [11]. Jasuja et al. have shown that the choice of the moieties substituted to the linker of an isostructural MOF series can have a tremendous impact on the adsorption capacity of water and also on the kinetic stability of the MOF structure [12].

On the other hand, sorption of water vapor can be a powerful tool for the detailed characterization of ultramicroporous materials at least on a qualitative basis. Commonly, the sorptives nitrogen (N_2_) and argon (Ar), at their respective boiling points (77.4 K and 87.3 K), are used for the purpose of material characterization. One major disadvantage is that these conventional adsorptives may not be able to enter the smallest micropores at cryogenic temperatures. As an alternative option carbon dioxide (CO_2_) isotherms at 273.15 K were introduced for a better resolution in the micropore range due to the higher kinetic activity at elevated temperatures. The suitability of this alternative procedure for the characterization of microporous materials has been repeatedly tested and validated by comparison to Ar and N_2_ measurements [13,14,15]. Due to their lower kinetic diameter compared to N_2_, Ar and CO_2_ (see Table 1), water molecules can penetrate smaller pores, which makes them a versatile probe for a wide range of materials [16,17]. Thus, under certain circumstances sorption experiments with water vapor can cover more textural and morphological information than with the conventional adsorptives [18]. This work aims to investigate the influence of different functional groups and transition metals of an isoreticular series of MOFs, named Imidazolate Frameworks Potsdam (IFP), on their inner texture, morphology and stability by sorption experiments with water vapor at 303.15 K. The interpretation of water sorption data is compared to N_2_ and CO_2_ sorption data and validated.

## 2. Methods and Materials

### 2.1. X-ray Diffraction Patterns

Powder X-ray diffraction (PXRD) patterns were measured on a Siemens Diffractometer D5005 (Munich, Germany) in Bragg-Brentano reflection geometry. The diffractometer was equipped with a copper tube, a scintillation counter and automatically incident- and diffracted-beam Soller slits. The generator was set to 40 kV and 40 mA. All measurements were performed with sample rotation. Data were collected digitally from 5° to 70° 2ϑ using a step size of 0.02° 2ϑ and a count time of 4 s per step. The simulated powder pattern for IFP-1 was calculated using single-crystal X-ray diffraction data and processed by the free Mercury software program provided by the Cambridge Crystallographic Data Center.

### 2.2. Particle Size Distributions

The particle size distributions of all samples used in this research project were determined with an optical measuring instrument (HORIBA LA-950, Retsch Technology, Haan, Germany), which is based on the principle of light scattering, applying the Mie theory, with a measurement range from 30 nm to 3 mm. To investigate the influence of aggregation and agglomeration, the samples were analyzed both in the untreated state (as synthesized) and after dispersion of the sample by using a dispersion agent as well as ultrasonic treatment and stirring. A dispersion agent Dispex AA4040 (BASF, Ludwigshafen, Germany) was used, which is a solution of an ammonium salt of an acrylic polymer in water.

### 2.3. Nitrogen and Carbon Dioxide Isotherms

The specific surface area, the pore size distribution and the pore volume of the microporous IFP-samples were derived from adsorption isotherms with the adsorptives nitrogen (N_2_) at 77.4 K and carbon dioxide (CO_2_) at 273.15 K, respectively, according to the IUPAC technical report for mesoporous and microporous materials [19]. Both adsorptives were purchased from Air Liquide with a purity of over 99.995 percent. The isotherms were recorded with a volumetric measuring device (NOVA 2000e, Quantachrome, Boynton Beach, FL, USA) in a pressure range of 0.001–1000 hPa. Prior to the measurement, an activation step was conducted by treating the sample at elevated temperatures (*T* ≈ 453.15 K) under ultrahigh vacuum (*p* ≈ 10^−4^ Pa). The specific apparent surface area was determined using the standardized Brunauer-Emmett-Teller method (BET method, [20]) while the pore size distribution was determined by applying the non-local density functional theory (NLDFT) for slit shaped carbon pores [21,22]. The authors emphasize that the derived quantities (pore volume and specific surface area) are not to be understood as absolute values that reflect factual reality, but are subject to statistical uncertainties due to the non-ideality of the system under investigation due to deviations from underlying simplifying assumptions, such as the formation of monolayers in the adsorbate phase or the geometrical shape of the adsorptives as ideal (point-symmetrical) spheres, which excludes a random arrangement of the latter. However, the derived values are quite suitable for quantitative comparison of the adsorptives among themselves, especially when they share the same topology.

### 2.4. Water Isotherms

#### 2.4.1. Experimental Setup

The adsorption isotherms for water vapor were recorded with a static gravimetric analyzer. The integrated precision balance with a resolution of 10^−7^ g at a sample quantity of approximately 100 mg allows the recording of the adsorbent load for each pressure stage (executed) until thermodynamic equilibrium is reached. As termination criterion, it was assumed that the equilibrium condition is fulfilled as soon as the weight gain due to adsorption is less than 10^−7^ g per minute for a time period of at least 20 min. To avoid condensation in the piping system of the plant, the entire system is located in a temperature-controlled housing. The sample is activated and regenerated in-situ with an electric furnace (*T* ≈ 453.15 K) and under ultrahigh vacuum (*p* ≈ 10^−4^ Pa) prior to the experiment. During the experiment the temperature is controlled with a circulation thermostat with integrated Peltier technology (Loop L 250, Lauda, Lauda-Königshofen, Germany). The pure water vapor source was generated by flashing the dissolved air from a reservoir filled with deionized water. Three isothermal cycles were run for each sample to ensure reproducibility of results. All samples were analyzed by PXRD and N_2_ before and after the water vapor measurements in order to trace material changes.

#### 2.4.2. Theory and Model

Since there is a deviation between the adsorption and desorption branch during the sorption of water vapor on meso- and microporous materials, also referred to as “hysteresis”, this circumstance must be taken into account for the isotherm modelling. The phenomenon of hysteresis between the adsorption and desorption branch can mainly be explained by terms of capillary condensation. With increasingly small pore radii ($r_{p}$), the interactions between the pore wall and adsorbate increase, which reduces the relative vapor pressure ($p_{i}^{sat}$) of the latter accordingly. This physical relation is expressed by the Kelvin equation (Equation (1)) with the surface tension ($\gamma_{i}$), the molar volume ($v_{i}$) and the contact angle of the condensed phase ($\vartheta_{i}$), the universal gas constant (R) and the absolutetemperature (T) [23]:

(1) $$ln\;\varphi_{i}=ln\frac{p_{i}}{p_{i}^{sat}}=-\frac{2\;\gamma_{i}\;v_{i}\cos\vartheta_{i}}{r_{p}\;R\;T}$$

As a result, smaller capillaries empty and fill at lower partial pressures than larger ones. In an irregularly arranged pore structure small pores can be placed upstream of larger ones. This has no impact on the adsorption process when pores are getting filled. However, it may have a certain impact on the desorption process when pores are getting emptied, as upstream small cavities may block the access to subsequent larger pore volumes and thus, the critical partial pressure for emptying can be limiting for the pore complex under consideration [24]. For more detailed insights into this “network theory” the reader is referred to further literature [10,25,26,27,28,29,30,31].

The convention stipulates that for water vapor the load is applied as a function of the relative humidity, which is generally described by the ratio of the partial pressure and the saturation vapor pressure of the considered species with values between 0 and 1:

(2) $$\varphi_{i}=\frac{p_{i}}{p_{i}^{sat}}$$

**Figure A1:**
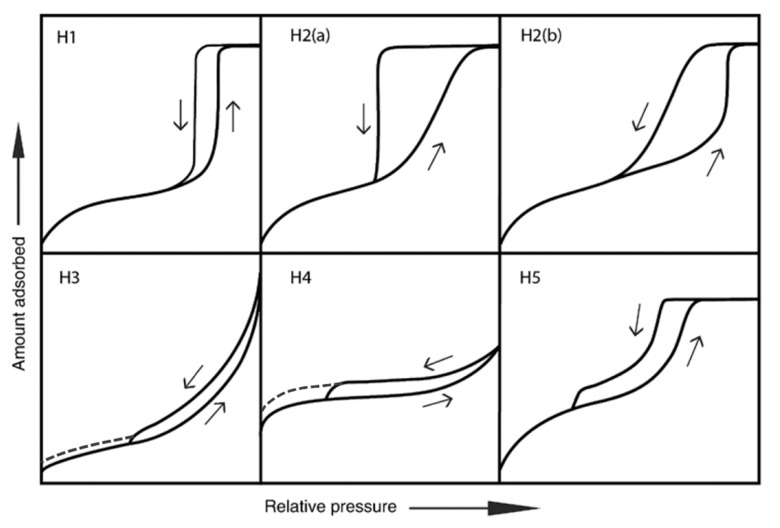
Classification of hysteresis exhibiting isotherms according to IUPAC [19,32]. The right-facing arrow indicates the adsorption branch and the left-facing arrow indicates the desorption branch.

According to the International Union of Pure and Applied Chemistry (IUPAC), hysteresis exhibiting isotherms are qualitatively mainly to be subdivided into six different types depending on their shape (see Appendix A, Figure A1 [19,32]).

The proposed isotherm model basically consists of two terms (see Equation (3)). One term ($n_{s}\left(\varphi \right)$) considers the uptake of water vapor by the functional groups of the adsorbent and the successive clustering of water molecules around these active sites. The other term ($n_{\mu }\left(\varphi \right)$) accounts for the water uptake by capillary condensation especially at elevated relative humidity. In this work the Guggenheim-Anderson-de Boer isotherm model (GAB) [33,34], $n_{s}\left(\varphi \right)$, was extended by adding the capillary condensation term from the model introduced by Do and Do [35], $n_{\mu }\left(\varphi \right)$. The GAB isotherm model represents a modification of the isotherm model by Brunauer, Emmett, Teller (BET). While the BET model distinguishes between two different types of layers, which are assigned to different energetic interaction levels between the adsorbent and the adsorbate, that is, the first layer where the heat of adsorption ($\Delta h_{1}^{ads}$) exceeds the enthalpy of vaporization ($\Delta h^{LV}$), and the subsequent layers where the heat of adsorption equals the enthalpy of vaporization, the GAB model accounts for an additional interlayer between the adsorbate layers 2 and 9 where the heat of adsorption attains intermediate values ($\Delta h_{1}^{ads}$ > $\Delta h_{2-9}^{ads}$ > $\Delta h^{LV}$).

(3) $$n\left(\varphi \right)=n_{s}\left(\varphi \right)+n_{\mu }\left(\varphi \right)=\frac{n^{\infty }\;c_{s}\;k_{s}\;\varphi }{\left(1-k_{s}\;\varphi \right)\left(1+\left(c_{s}-1\right)\;k_{s}\;\varphi \right)}+\frac{n_{\mu }^{\infty }\;K_{\mu }\;\varphi^{m}}{1+K_{\mu }\;\varphi^{m}}$$

with the dimensionless rate constants:

(4) $$c_{s}=\frac{a_{1}\;b_{2}}{b_{1}\;a_{2}}\exp\left(\frac{\Delta h_{1}^{ads}-\Delta h^{LV}}{RT}\right)$$

and

(5) $$k_{s}=\exp\left(\frac{\Delta h_{2-9}^{ads}-\Delta h^{LV}}{RT}\right)$$

$n^{\infty }$ and $n_{\mu }^{\infty }\;$are the saturation capacities for the cluster term (GAB) and the capillary condensation term (Do & Do), respectively. The latter of which is described by a Langmuir-like model with the equilibrium constant, $K_{\mu }$, and the average number of water molecules comprising a cluster, m. The model equation was approximated to experimental data using the nonlinear least squares method by applying the Levenberg-Marquardt algorithm [36].

### 2.5. Material Synthesis and Characteristics

All chemicals used for material synthesis were purchased from commercial suppliers (Sigma-Aldrich, St. Louis, MO, USA; Fluka, Buchs, Switzerland and Alfa Aesar, Ward Hill, MA, USA) and used without further purification. The syntheses of the IFP-species were previously reported by Holdt et al. and reproduced according to the published procedures [37].

**Figure 1:**
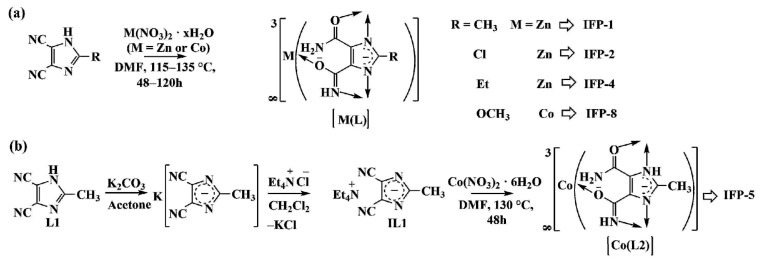
General synthetic strategies for IFP-1, -2, -4 and -8 (**a**) and IFP-5 (**b**), [39,40,41].

The IFP structure is similar to that of zeolitic imidazolate frameworks (ZIF, [38]) whereas the main difference relies on the coordination of their particular metal center. The metal ions of the IFPs are—unlike the tetracoordinated ZIFs—pentacoordinated by donor atoms of three ligands forming a distorted environment with a trigonal-bipyramidal geometry. In detail, the IFP structures are built from 2-substituted imidazolate-4-amide-5-imidates (L), that are generated in situ by partial hydrolysis of 4,5-dicyano-2-substituted imidazole (L1) under solvothermal conditions in dimethylformamide (DMF) and in the presence of a metal nitrate hydrate salt, with the exception of IFP-5, for which the ligand (L1) is provided as an anionic linker precursor (IL1, see Figure 1b).

**Figure 2:**
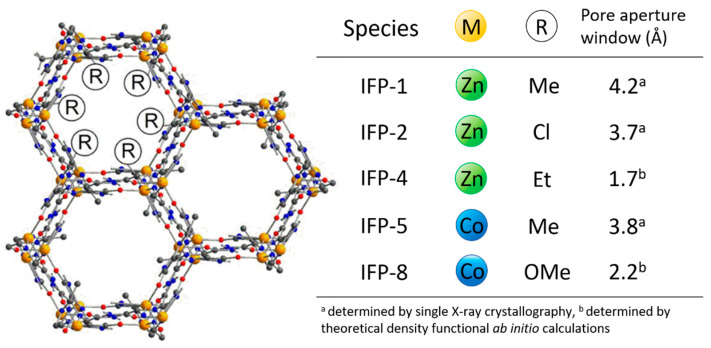
Schematic presentation of the 1D hexagonal channels of IFPs (**left**) (metal center: yellow, nitrogen: blue, oxygen: red, carbon: grey) with an overview of the corresponding pore aperture windows derived from crystallographic data and density functional ab initio calculations (**right**).

The general chemical formula is given as [M(C_5_H_3_N_4_O_2_)-R] with the transition metal center, M, and the substituent R, at the C2 position of the amide-imidate-imidazolate (C_5_H_3_N_4_O_2_) linker (for IFP-1: M = Zn, R = CH_3_; IFP-2: M = Zn, R = Cl; IFP-4: M = Zn, R = C_2_H_5_; IFP-5: M = Co, R = CH_3_; IFP-8: M = Co, R = OCH_3_, [39,40,41], see Figure 2).

**Figure 3:**
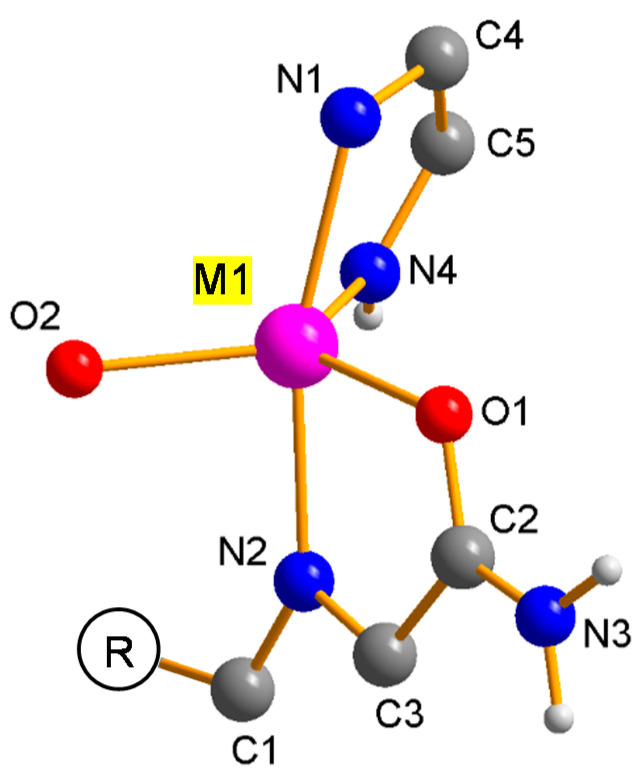
Asymmetric unit of isostructural IFPs in general, showing the coordination environment of the metal center and the bridging mode of inter-connecting Linker L.

**Figure 4:**
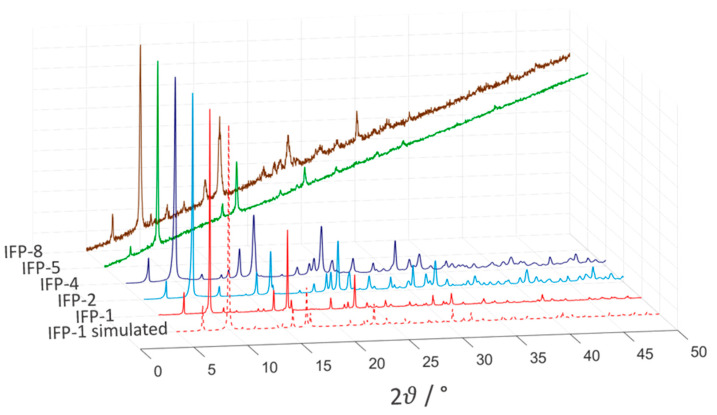
Normalized powder X-ray diffractograms of all the considered IFPs (as synthesized).

In short, imidates N4 and O2 and amides O1 reside in equatorial positions and two imidazolate N atoms (N1 and N2) occupy the axial positions (see Figure 3). This five-fold coordination leads to an M^2+^ center with Lewis acid properties. The structure of the IFPs have been previously resolved by X-ray crystallographic analyses as well as density functional ab initio calculations and confirmed by IR and NMR spectroscopy [37]. In situ functionalized ligands linked with M^2+^ ions form the neutral microporous imidazolate MOF with 1-D hexagonal channels (see Figure 2). M^2+^ ions at IFP structure and bridging ligands act as 3-connected topological species forming a net with a uninodal etb-topology [39,42]. The functional groups at the C1 atom of the linker protrude into the open channels, tuning the pore aperture window (1.7–4.2 Å), polarity and functionality of the channel walls (see Figure 2). The experimentally determined PXRD patterns of the IFPs showed a positive agreement with the corresponding simulated one for IFP-1 as well as with one another, showing characteristic peaks at the same rotation angles (see Figure 4). This clearly proves that the IFPs represent an isoreticular series of MOFs with identical topology. The baseline drift for IFP-5 and -8 is due to the secondary fluorescence of the Co center under X-ray excitation by the copper anode [43,44].

By varying the metal centers or the substituents of the linker molecules protruding into the open channels different pore sizes and physicochemical properties of the obtained one-dimensional channel structures are achieved. This allows to solely investigate the impact of the mentioned modifications onto the characteristics of these porous materials while their topology remains unaltered.

## 3. Results and Discussion

**Figure 5:**
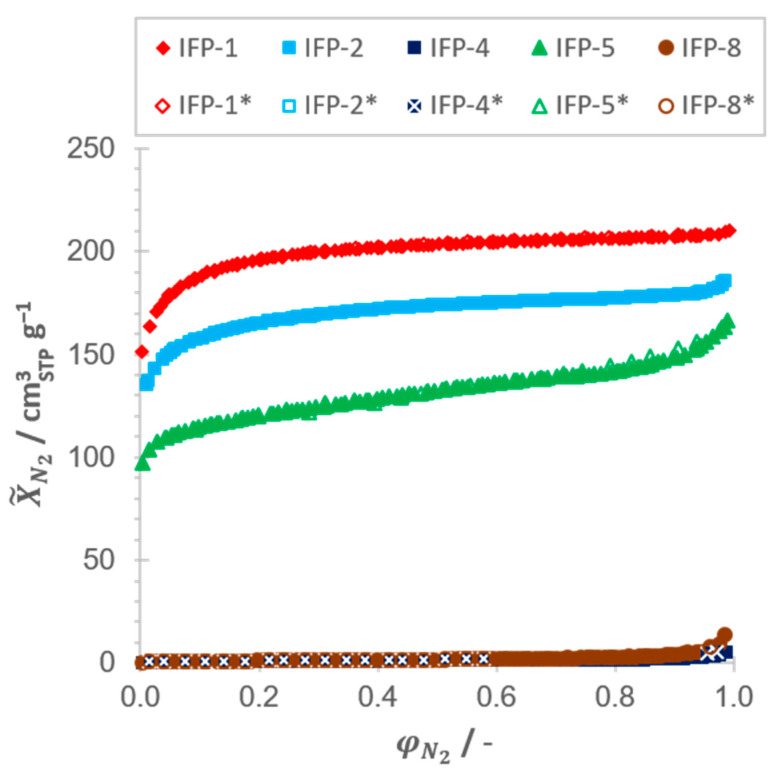
Adsorption and desorption (*) isotherms of N_2_ on all IFP samples at *T* = 77.4 K.

**Figure 6:**
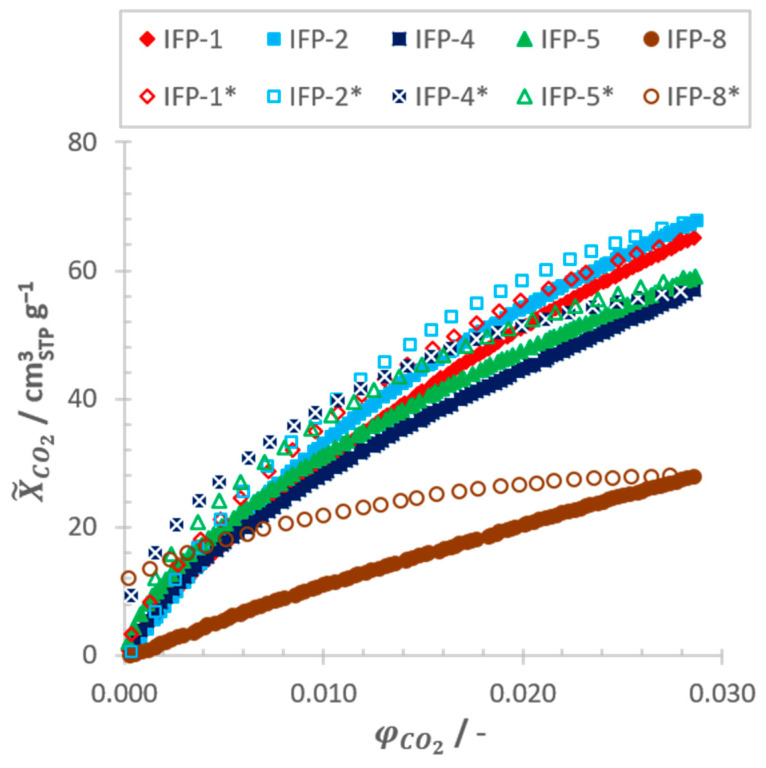
Adsorption and desorption (*) isotherms of CO_2_ on all IFP samples at *T* = 273.15 K.

Although N_2_ isotherms at 77.4 K (see Figure 5) are the common method for the characterization of porous materials, it is limited with respect to resolution in the ultramicroporous range as well as macropores bigger than 350 nm in diameter [45,46]. For this purpose, CO_2_ isotherms at 273.15 K (see Figure 6) were also used to resolve the micropores. CO_2_ not only has a smaller kinetic diameter ($d_{K}$) in principle (see Table 1), but is also kinetically more active at an experimental temperature of 273.15 K compared to N_2_ at 77.4 K and therefore penetrates micropores more easily. Another reason for the better resolution of the micropore range is given due to a temperature-related flexibility of the adsorbents used, as it has often been reported for MOFs [47,48,49,50,51] as well as for IFP compounds [52,53]. However, isotherms derived from water vapor, which exhibits the smallest kinetic diameter of the selected adsorptives, allow a better insight in local phenomena.

**Figure 7:**
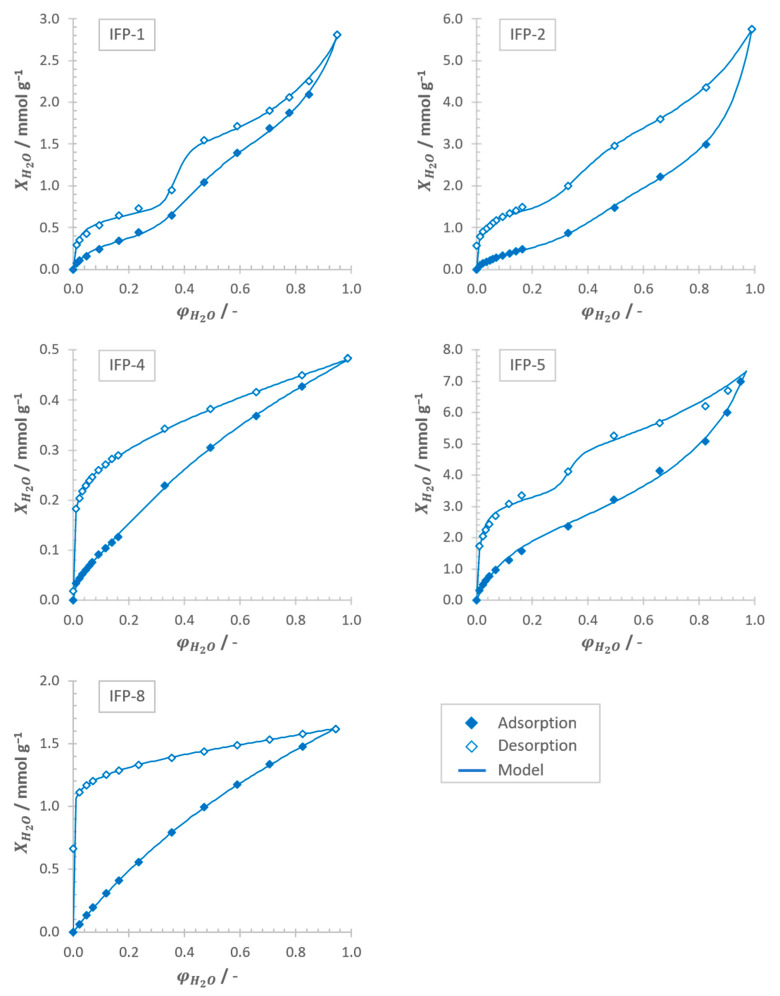
Adsorption (full diamonds ◆) and desorption (empty diamonds ◇) equilibria of water vapor on all IFP samples at *T* = 303.15 K approximated by the model equation (straight line ***—***).

Figure 7 shows the experimental adsorption and desorption equilibria of IFP-1, -2, -4, -5 and -8 at 303.15 K approximated by the model equation introduced above (see Equation (3)). What all isotherms have in common is a distinct hysteresis loop between the adsorption and desorption branch over the entire range of moisture. The phenomenon of low pressure hysteresis down to the lowest attainable pressures, which is most significant for IFP-4 and -8 is a strong indicator for highly microporous ($d_{p}<$ 2 nm) up to ultramicroporous ($d_{p}<$ 0.7 nm) systems [19,32]. Fully sample regeneration is only possible if the adsorbent is outgassed at higher temperatures. This characteristic feature may occur due to the swelling of non-rigid pore structures, which is known for several microporous MOF species [48,54], or due to irreversible uptake by chemisorption [19,32]. However, a fundamental difference can be observed regarding the shape of the isotherms. While IFP-1, IFP-2 and IFP-5 exhibit an H3-type behavior (see Appendix A, Figure A1) according to the IUPAC classification [32], IFP-4 and IFP-8 are attributable to the H4-type (see Appendix A, Figure A1), but without showing the trend towards unlimited adsorption capacities at high relative humidity ($\varphi_{H_{2}O}\to$ 1).

**Figure A2:**
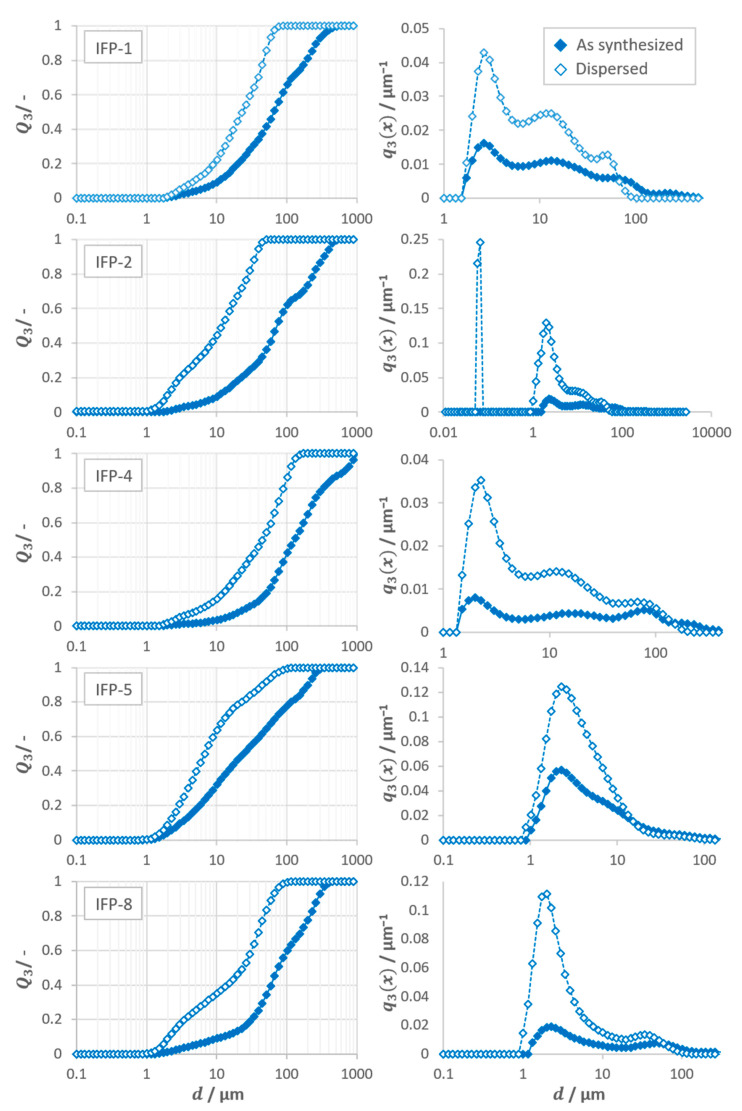
Volume sum distribution $Q_{3}\left(x\right)$ (**left** column) and volume density distribution $q_{3}\left(x\right)$ (**right** column) of all IFP samples before (full diamonds ◆) and after dispersion (empty diamonds ◇).

Both types, H3 and H4, have in common that they are primarily observed on aggregate or agglomerate forming plate-like particles, which are giving rise to the assumption of the presence of slit-shaped pores, which was already concluded from previous investigations [40,52,53]. Comparison of the particle size analysis of the untreated as synthesized samples and the dispersed samples are validating this postulate. Thus, the particle size distributions—volume density distribution, $q_{3}$ (see Appendix A, Figure A2, right column), and volume sum distribution, $Q_{3}$ (see Appendix A, Figure A2, left column)—of each sample were significantly shifted towards smaller particle sizes after breaking the agglomerates and aggregates by employing the above mentioned dispersion methods (see Section 2.2). A quantitative evaluation of the results is possible by comparison of the median diameters (see Appendix A, Table A1). For IFP-1, -4, -5 and -8, the median diameters were reduced by the factor of about 3, for IFP-2 even by the factor of almost 6. This becomes even more comprehensible in view of the volume density distribution of IFP-2, since here a completely new, very small particle fraction with a size between 50 and 100 nm is detected after dispersion.

**Figure A3:**
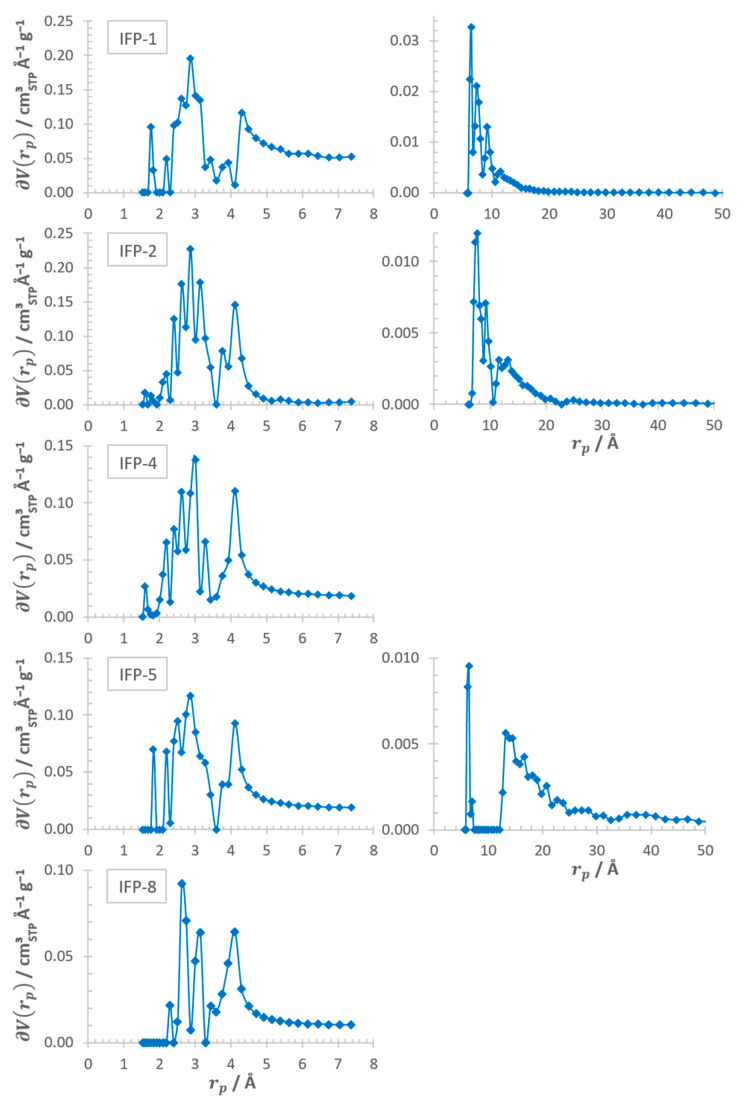
Pore size distribution of all IFP samples derived by means of NLDFT from isotherms recorded with CO_2_ (**left**) and N_2_ (**right**, IFP-4 and -8 are missing because of irrelevance).

The H3 type with three significant inflection points, which are particularly pronounced in the desorption branch, indicates the coexistence of two dominant pore sizes in the ultramicropore and supermicro-/mesopore range, which are emptying at $\varphi_{H_{2}O}\to \;$0 and $\varphi_{H_{2}O}\approx$ 0.3–0.4, respectively. This is in good accordance to the pore size distributions derived from isotherm measurements taken with the adsorptives CO_2_ and N_2_. For IFP-1, -2 and -5, significant amounts of N_2_ as well as CO_2_ were adsorbed at 77.4 K and 273.15 K, respectively (see Figure 5; Figure 6). The pore size distributions (see Appendix A, Figure A3) derived from these measurements by means of NLDFT yield a cumulative pore volume that is shared between micro- ($d_{p}\leq \;$2 nm) and mesopores (2 nm $<d_{p}\leq$ 50 nm), with a clear predominance of micropores for all three adsorbents under consideration (see Table 2). This significant deviation of the pore size distributions compared to simulated accessible pore diameters derived from the crystallographic data (see Figure 2) can only be attributed to the formation of defects in the crystal structure during material synthesis, which were already known for various MOFs [55] and also for IFPs themselves [56,57].

The H4 type, which is equal to the shape of a type I isotherm according to IUPAC, is characteristic for adsorbents that contain micropores solely and shows again good agreement to the conclusions to be drawn from N_2_ and CO_2_ measurements. Since the measurements with N_2_ show no uptake capacity for both samples—IFP-4 and -8—(see Figure 5), but merely for CO_2_ (see Figure 6), the presence of macro- and especially mesopores can be excluded. Therefore, water vapor isotherms are exhibiting no inflection points in this case. Although only micropores are detected for IFP-4 and -8, it must also be stated that the experimentally determined pore diameters exceed the theoretical values derived from the crystallographic data, since the kinetic diameter of all used adsorptives is larger. Again, only flexibility and gate opening effects or defects in crystal structure of the IFP samples can be considered as cause for this deviation. In fact, previous publications have already referred to the flexible properties of ethyl- and methoxy-substituted linkers of IFP-4 [53] and -8 [52], respectively.

The reason why the H3 type isotherms (IFP-1, -2 and -5), in contrast to the H4 type isotherms (IFP-4 and -8), appear to tend towards unlimited adsorption capacities at high relative humidity ($\varphi_{H_{2}O}\to 1$) is attributed to the presence of either intra- or interparticle macro-/mesopores [19]. As already seen from the N_2_ measurements for IFP-1, -2 and -5, defects in the metal framework might lead to the formation of intraparticle meso- and macropores. In addition, interparticle macropores can also be present by a special crystal morphology due to aggregate formation or properties of the particle bed itself. The latter, however, seems to be most probable and can be explained by the median values of the particle size distributions, which are lowest for IFP-1, -2 and -5 (see Appendix A, Table A1), allowing for the highest bulk densities and lowest interparticle voids, respectively.

These observations are also in good agreement with the conclusions of Sircar’s theory for capillary condensation of vapors on porous materials [31]. According to his theory isotherms taken on adsorbents with high surface area to pore volume ratios ($a/V_{p}$) are appoaching type I (or H4) shapes, while in case of lower surface area to pore volume ratios type IV (or H3) is approached. As shown in Table 2 the surface area to pore volume ratio for IFP-4 and -8 (H4) is significantly higher compared to IFP-1, -2 and -5, which is convincing as the pore volume decreases exponentially stronger than the surface area with decreasing pore diameter.

The fact that CO_2_ isotherms are, in contrast to N_2_, exhibiting a hysteresis behavior can either be explained by stronger adsorbent-adsorbate interactions due to the higher polarizability ($\alpha_{CO_{2}}\;$= 2.34 Å^3^, $\alpha_{N_{2}}\;$= 1.05 Å^3^, [58]) and quadrupole momentum ($q_{CO_{2}}\;$= −16.94·10^−20^ C Å^2^, $q_{N_{2}}\;$= −4.47·10^−20^ C Å^2^, [58]) of CO_2_ or related to network effects in the micropore range of the IPF-samples.

Table 2 also lists the maximum water loads of the examined IFP samples in order to draw conclusions about the influence of the different moieties of the linkers and transition metals employed. For IFP-1, which has Zn as transition metal center and a methyl-group as moiety connected to the C2 position of the imidazolate linker, the maximum load at 273.15 K is approximately 2.81 mmol g^−1^. By substituting the methyl group with chloride (IFP-2), the maximum load increases by over 100% to 5.75 mmol g^−1^. Thus, the adsorption capacity of water vapor can be significantly increased by the introduction of a polar C-Cl bond, but with only a slightly lower surface area. However, if the methyl residue of IFP-1 is extended by methylene unit (IFP-4) the adsorption capacity collapses almost by the factor of 6 to 0.48 mmol g^−1^. This can be explained by the reduction of the pore diameter and the specific surface area as well as the increase of the non-polar character of the channel walls (see Table 2 and Appendix A, Figure A3). If—again starting from IFP-1—the transition metal center Zn is replaced by Co (IFP-5), the adsorption capacity can even be further increased by 150% to 6.99 mmol g^−1^. Apparently, the substitution of Zn by Co causes an increase in hydrophilicity and an increase in the cluster density of water molecules, compensating for the lower specific surface area and pore volume compared to IFP-1. This claim is also supported by the isotherm model, in which the cluster size of water molecules, m, increases from ~6 for IFP-1 also by 150% to ~15 for IFP-5 (see Appendix A, Table A2). Under consideration of the electron configuration of both transition metals it seems reasonable since Co^2+^ ([Ar] 3d^7^) in contrast to Zn^2+^ ([Ar] 3d^10^) exhibits an incompletely electron-filled d-shell, which polarize the channel walls and is also displayed by electronegativity on the Pauling scale ($\chi_{Co}$ = 1.88 > $\chi_{Zn}$ = 1.65, [59]). Substitution of the methyl group by the sterically more demanding methoxy group (IFP-8) leads in turn to a reduction in capacity by the factor of 4 to 1.6 mmol g^−1^ as the pore volume and the specific surface decrease drastically. The cluster size of water molecules, however, appears to increase further since the methoxy group raises the polarity of the channel walls, forming an ether unit.

As mentioned before, the pore volumes cannot be determined reliably from the measurements with N_2_ and CO_2_ in their entirety, since on the one hand a large part of the pore volume is inaccessible to the N_2_ molecule because it is blocked by micropores and on the other hand because CO_2_ at 273.15 K is only measured up to 0.1 MPa—which is far below its saturation vapor pressure of 3.49 MPa—due to plant limitations. Thus, only the micropore range could be resolved with the CO_2_ measurements accordingly. Based on the maximum loads achieved from experiments with water vapor, the pore volumes (see Table 2) were estimated assuming complete filling of the pore system of the IFP samples and an adsorbate density corresponding to the density of water at liquid state at the same temperature. With the exception of IFP-4, the pore volumes calculated in this way are significantly larger than those estimated from the measurements with N_2_ and CO_2_. In particular, the apparent pore volumes of the most polar adsorbents IFP-2, -5 and -8 determined from adsorption with water vapor exceed those determined by N_2_ and CO_2_ by a factor of three to five. In addition to the strong polarity—due to the C-Cl bond of IFP-2 and the polarizable Co centers of IFP-5 and -8—this can also be attributed to a better resolution of the micropore range by employing the adsorptive H_2_O, which is quite smaller than CO_2_ in terms of its kinetic diameter (see Table 2). The latter also explains the 25% increase in pore volume for IFP-1 with its non-polar methyl group. Only the apparent pore volume of IFP-4 is reduced by half. This may be due to a bridging effect of the water molecules around the strongly non-polar and sterically demanding ethyl group at its imidazolate linker [18,60].

The shown water uptakes for the IFP samples are comparable to those reported for zeolitic imidazolate frameworks, ZIF-8 (0.5 mmol g^−1^) and ZIF-90 (18 mmol g^−1^) at 308 K [61,62]. The imidazolate linkers used in ZIF-8 and ZIF-90 are 2-methylimidazolate and imidazolate-2-carboxyaldehyde. ZIF-8 exhibits low water uptake demonstrating its framework hydrophobicity. Due to the presence of the hydrophilic carbonyl group in the linker of ZIF-90 a large enhancement in water sorption capacity occurs. The water vapor uptake of the IFPs can be influenced by the substituent R in the position 2 of the amide-imidate-imidazolate linker too. Moreover it is also shown that the node—Zn or Co(II)—has an impact on the water vapor uptake, however, in comparison to a lot of other MOFs the IFPs have to be classified as hydrophobic porous coordination polymers too [10,11].

**Figure 8:**
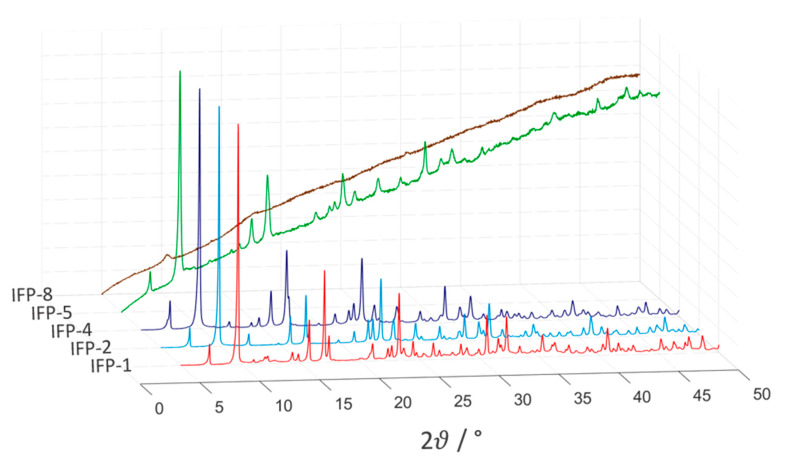
Normalized powder X-ray diffractograms of all IFP samples after water uptake experiments.

All IFP samples show reproducible water uptake during the three isotherm cycle runs. However, the PXRD analyses performed after the isothermal cycles indicate that IFP-8 loses its crystalline structure and is degraded to an amorphous substance (see Figure 8), while IFP-1, -2, -4 and -5 maintain their structure. This can be related to the −I-effect induced by the methoxy group of IFP-8 and the polarizable effect of the Co center which enhance the tendency to a nucleophilic attack on the C2 atom of the imidazolate linker, leading to destabilization of the crystal structure. The BET surface area of IFP-8 also collapses by 80%, while it remains approximately constant for the other IFPs. Therefore, the material degradation must have occurred either during the first cycle or after the last one. In the latter case, recontamination by atmospheric oxygen or long-term effects could be decisive.

## 4. Conclusions

An isostructural series of IFPs with different functional properties but identical topology was investigated for its characteristic sorption properties with water vapor (at 303.15 K), N_2_ (at 77.4 K) and CO_2_ (at 273.15 K). In general, it could be shown that the qualitative analysis of water vapor sorption measurements has good agreement with the evaluations drawn from the established measuring methods with N_2_ and CO_2_. All sorption measurements with water vapor show a strong hysteresis, which indicates a dominance of microporous networks on the one hand and is especially documented for powders forming agglomerates or aggregates on the other hand. The latter was confirmed after dispersion of the samples by determining particle size distributions.

The maximum water uptake is in the range of 0.48 mmol g^−1^ (IFP-4) to 6.99 mmol g^−1^ (IFP-5) and similar to those reported for ZIFs. All IFPs showed reproducible results for three isotherm cycles. However, PXRD measurements and BET analysis revealed that none of the samples are degraded by water vapor sorption, with the exception of IFP-8, which degraded to an amorphous substance, losing 80% of its BET surface area. Thus, material decomposition must have started after the isotherm cycles with water vapor due to recontamination with atmospheric oxygen or long-term effects.

The water isotherms are approximated with a combined model, by extending the GAB model with the capillary condensation term of the Do and Do model. Comparison of IFPs with the same transition metal Zn among themselves shows that the highest water vapor adsorption capacities were achieved with the polar linker substituent chloride (IFP-2), followed by the less polar methyl (IFP-1) and ethyl (IFP-4), consecutively. The substitution of the transition metal Zn (IFP-1) by Co (IFP-5) with the same linker moiety (methyl) also leads to a significant increase in uptake capacity due to the higher channel wall polarizability induced by the Co entre. However, the substitution of the methyl group by the sterically more demanding methoxy group leads to a collapse of the adsorption capacity in analogy to IFP-4 with its ethyl group. Although the theoretically accessible pore diameters for IFP-4 and -8 are significantly smaller than the kinetic diameters of all adsorptives employed, significant adsorption capacities were found at least for water vapor and CO_2_, which is an indicator for the flexibility of the substituent R. Both species are exhibiting H4 (type I like) behavior for adsorption of water vapor, which clearly indicates the exclusive presence of micropores and is confirmed by the CO_2_ and N_2_ measurements. IFP-1, -2 and -5 are exhibiting an H3 (type IV like) behavior with three distinct inflection points, which is indicative for the presence of all pore sizes (micro-, meso- and macropores).

In a nutshell, it can be stated that the information derived from the water vapor sorption measurements exceeds the collective information content of the CO_2_ and N_2_ measurements on a qualitative level.

## Figures and Tables

**Table 1:** Molecular weight (M) and kinetic diameter ($d_{K}$) of the adsorptives used for adsorption experiments (water, carbon dioxide, nitrogen, [17]) and for the common adsorptive argon [16].

Species	M (g mol^−1^)	$d_{K}\;(Å)$
H_2_O	18	2.65
CO_2_	44	3.30
Ar	40	3.40
N_2_	28	3.64

**Table 2:** Overview of the characteristic data of the tested IFP samples gained from isotherms with N_2_, CO_2_ and water vapor: Specific surface (a), micro pore volume ($v_{p,\mu }$), overall pore volume ($v_{p,\Sigma }$), specific surface to pore volume ratio ($a/v_{p}$), maximum uptake of water vapor at *T* = 303.15 K ($X_{H_{2}O,\;max}$) and total pore volume derived from maximum uptake of water vapor ($v_{p,\Sigma }^{\prime }$).

Species	Isotherm Type	a (m^2^ g^−1^)	$v_{p,\mu }\;({\mathrm{cm}}^{3}\;g^{-1})$	$v_{p,\;\Sigma }\;({\mathrm{cm}}^{3}\;g^{-1})$	$a/v_{p,\Sigma }\;(m^{2}\;{\mathrm{cm}}^{-3})$	$X_{\;H_{2}O,\;max}\;(\mathrm{mmol}\;g^{-1})$	$v_{p,\Sigma }^{\prime }\;({\mathrm{cm}}^{3}\;g^{-1})$
IFP-1	H3	800 ^a^	0.38 ^b,c^	0.40 ^b,c^	2000	2.81	0.51 ^d^
IFP-2	H3	777 ^a^	0.28 ^b,c^	0.31 ^b,c^	2507	5.75	1.03 ^d^
IFP-4	H4	562 ^a^	0.17 ^b,c^	0.17 ^b,c^	3306	0.48	0.09 ^d^
IFP-5	H3	647 ^a^	0.22 ^b,c^	0.27 ^b,c^	2396	6.99	1.27 ^d^
IFP-8	H4	233 ^a^	0.06 ^b,c^	0.06 ^b,c^	3888	1.62	0.29 ^d^

^a^ derived from adsorption isotherm with CO_2_ at 273.15 K by applying the BET-method, ^b^ derived from adsorption isotherm with CO_2_ at 273.15 K by applying NLDFT, ^c^ derived from adsorption isotherm with N_2_ at 77.4 K by applying NLDFT, ^d^ calculated from the maximum load of water, $X_{H_{2}O,\;max}$, assuming an adsorbate density equal to the density of water at liquid state.

## Glossary

1. **Latin Symbols**

**Symbol**	**Meaning**	**Units**
a	specific surface/rate constant	m^2^ g^−1^/-
b	rate constant	-
c	rate constant	-
d	diameter	m
$\Delta h_{1}^{ads}$	enthalpy of adsorption 1st layer	J mol^−1^
$\Delta h_{2-9}^{ads}$	enthalpy of adsorption 2nd to 9th layer	J mol^−1^
$\Delta h_{}^{LV}$	enthalpy of vaporization	J mol^−1^
k	equilibrium constant	-
K	equilibrium constant	-
m	amount of molecules per cluster	-
M	molar mass	g mol^−1^
n	number of moles	mol
p	pressure	Pa
$p_{}^{sat}$	saturation vapor pressure	Pa
q	quadrupole momentum	C Å^2^
$q_{3}$	volume density distribution	m^−1^
$Q_{3}$	volume sum distribution	-
r	radius	mm
R	universal gas constant	J mol^−1^ K^−1^
T	absolute temperature	K
v	molar volume	m^3^ mol^−1^
$v_{p}$	pore volume	m^3^ g^−1^
X	molar loading (adsorbed phase)	mol g^−1^
$\tilde{X}$	volume loading (adsorbed phase)	m^3^ g^−1^

2. **Greek Symbols**

**Symbol**	**Description**	**Units**	
α	polarizability	Å^3^	
γ	surface tension	Nm^−1^	
ϑ	contact angle	rad	
φ	relative pressure	-	
χ	electronegativity on the Pauling scale	-	

3. **Subscripts**

**Symbol**	**Description**
∞	infinity
μ	capillary condensation term
max	maximum
s	cluster term
t	total
*i*	component i

4. **Abbreviations**

**Symbol**	**Description**
Ar	argon
BET	Brunauer-Emmett-Teller
Co	cobalt
CO_2_	carbon dioxide
GAB	Guggenheim-Anderson-de Boer
H_2_O	water
IFP	Imidazolate framework Potsdam
IL	ionized linker
IR	infrared
L	linker
M	metal center
MOF	metal organic framework
N_2_	nitrogen
NMR	nuclear magnetic resonance
PXRD	powder x-ray diffractometry
R	moiety
STP	standard temperature and pressure
ZIF	zeolitic imidazolate framework
Zn	zinc

## Appendix A

**Table A1:** Volumetric median diameters of the IFP samples before and after agglomerate breakage (*).

	IFP-1	IFP-2	IFP-4	IFP-5	IFP-8
$d_{50,3}$ (µm)	62.96	71.79	123.87	22.53	75.39
$d_{50,3}^{*}$ (µm)	23.52	12.59	48.56	7.24	28.08

**Table A2:** Numerical parameters for the equilibrium model applied for the adsorption and desorption (hysteresis loop) of water vapor on IFP samples at *T* = 303.15 K.

	IFP-1		IFP-2		IFP-4		IFP-5		IFP-8	
	ads	des	ads	des	ads	des	ads	des	ads	des
$n_{s}$	0.38	0.56	0.52	1.28	0.06	0.23	2.38	3.07	2.72	0.34
$k_{s}$	0.86	0.77	0.90	0.73	0.52	0.27	0.68	0.53	0.13	3.47
$c_{s}$	19.24	113.80	16.02	153.60	146.10	1031.00	13.59	193.60	8.29	0.40
$n_{\mu }$	0.78	0.72	1.20	1.24	0.55	0.27	4.26	1.03	0.21	2.64
$K_{\mu }$	100.70	1.0·10^3^	24.44	241.20	1.84	1.59	0.22	3.5·10^6^	0.04	0.45
m	5.62	7.11	4.27	5.74	1.40	1.07	14.93	13.76	59.72	60.22
